# Circulating GLP-1 Levels in Patients with Pheochromocytoma/Paraganglioma

**DOI:** 10.1155/2022/4203018

**Published:** 2022-12-14

**Authors:** Nan Chen, Yan Chen, Fenghua Lai, Li Chen, Rui Zeng, Ling Pei, Liting Wu, Chenxue Wang, Yanbing Li, Haipeng Xiao, Xiaopei Cao

**Affiliations:** Department of Endocrinology, The First Affiliated Hospital, Sun Yat-sen University, 58 Zhongshan 2nd Rd, Guangzhou 510080, China

## Abstract

The sympathoadrenal system has been shown to stimulate the secretory activity of enteroendocrine cells, although the response is transient. Our aim was to investigate the effects of long-term catecholamine excess on circulating glucagon-likepeptide-1 (GLP-1) levels in patients with pheochromocytoma/paraganglioma (PPGL). Thirty patients diagnosed with PPGL were analyzed. A significant negative association was observed between fasting plasma GLP-1 levels and elevated plasma-free metanephrine (*r* = −0.407, *p* = 0.026). After adjustment for age, sex, body mass index (BMI), serum creatinine, and the presence of hyperglycemia, the negative association between plasma GLP-1 and metanephrine persisted by multiple linear regression analysis (*β* = −0.493, *p* = 0.013). Positive correlations between fasting glucose and plasma metanephrine (*r* = 0.380, *p* = 0.038) and normetanephrine levels (*r* = 0.450, *p* = 0.013) were also found. Mean fasting levels of total GLP-1 increased significantly from 25.81 to 39.01 pmol/L (*p* = 0.017) after PPGL resection. In conclusion, long-term overproduction of catecholamines appears to induce suppression of GLP-1 production compared to an acute response to a stress stimulus. Further studies are required to elucidate the mechanism of GLP-1 secretion with chronic exposure to catecholamine.

## 1. Introduction

Pheochromocytoma and paraganglioma (PPGL) are catecholamine-producing neuroendocrine tumors arising from chromaffin tissue of the adrenal medulla and the extra-adrenal sympathetic paraganglia, respectively [1, 2]. PPGLs most often arise from the adrenal gland (pheochromocytoma, PCC), while approximately 15–20% occur at extra-adrenal sites (paraganglioma, PGL) [3, 4]. Functional PPGLs are known to produce catecholamines, mainly norepinephrine and epinephrine, and metabolize them to their O-methylated metabolites. Within the tumor, dopamine is metabolized to methoxytyramine, epinephrine to metanephrine (MN), and norepinephrine to normetanephrine (NMN) [5]. Levels of plasma-free metanephrines (MNs) are known to be stable [6]. Excessive release of catecholamines by these tumors gives rise to various signs and symptoms, such as chronic or paroxysmal hypertension, tachycardia, sweating, and headache, and can result in metabolic disorders and cardiovascular events [7–9]. Surgical resection of PPGL is the mainstay of treatment [10].

PPGLs, rare neuroendocrine tumors, represent a useful model for studying the effects of long-term catecholamine overproduction on metabolic or hormone disorders. According to studies, the prevalence of diabetes mellitus (DM) in PPGL patients has been reported to vary from 21 to 37% [11, 12]. It has been controversial whether the main factor of glucose intolerance is impaired insulin secretion or increased insulin resistance, and a definite mechanism remains to be further explored. Glucagon-likepeptide-1 (GLP-1), a peptide hormone, has been shown to exert a wide range of effects on glucose metabolism and cardiovascular function [13]. The sympathoadrenal system has been shown to stimulate the secretory activity of enteroendocrine cells, while the response to adrenergic activation is transient. Limited research suggests that epinephrine and norepinephrine can affect GLP-1 secretion directly from intestinal L cells [14–16]. These studies found that epinephrine increases GLP-1 secretion as a result of combined activation of the three types of receptors (*α*1-, *α*2-, and *β*1-adrenergic receptors), while norepinephrine inhibits GLP-1 secretion via its action on the *α*-adrenergic receptor. However, the chronic effect of catecholamine on GLP-1 secretion and the influence of long-term catecholamine activity at very high plasma concentrations in humans remain unknown. Recently, Petrák and coworkers found reduced postprandial levels of bioactive GLP-1 during a dynamic meal test in patients with PCC with an adrenergic biochemical phenotype (PHEO), but they did not find any relationship between plasma catecholamines or MNs and GLP-1 levels [17]. In type 2 diabetes (T2D), GLP-1 secretion has been suggested to be impaired. However, further studies have found that this defect appears to be a consequence of the diabetic state rather than a cause, and chronic hyperglycemia exposure can affect GLP-1 secretion [18–20]. Therefore, it is unclear whether impaired GLP-1 secretion in PPGL patients is caused by metabolic abnormalities or by catecholamine overdose. The postprandial plasma GLP-1 area under the curve was significantly lower in patients with a diabetic state than in those with normal glucose tolerance (NGT), while baseline levels of plasma GLP-1 in diabetic patients were higher than or not different from those in patients with NGT [21–23]. Therefore, evaluating changes in fasting circulating GLP-1 levels in PPGL patients may reduce the interference of metabolic risk factors compared with postprandial GLP-1. Thus, the aim of our study was to evaluate the relationship between basal circulating levels of GLP-1 and plasma MNs in PPGL, as well as changes in GLP-1 levels before and after tumor removal.

## 2. Methods

### 2.1. Study Participants and Data Collection

Thirty patients with PPGLs who were scheduled to undergo surgical resection at the First Affiliated Hospital, Sun Yat-sen University, were asked to participate in our study. In this article, we use the term “PGL” for paragangliomas of the abdomen, pelvis, and thorax. Head-neck paragangliomas (HNPGLs) are usually hormonally inactive and were not included. We collected data on age, sex, body mass index (BMI), waist circumference, past medical history, history of medication use, symptoms, tumor characteristics, relative hormones, and biochemical testing from all patients. In addition, patients were followed up after surgery, and plasma MN, NMN, and GLP-1 levels were measured. Postoperative samples were available for 13 patients. Reasons for not obtaining a postsurgical sample included the difficulty of patients returning to our medical department and refusal to have additional blood drawn. Hyperglycemia includes patients with either IFG (fasting plasma glucose 6.1–6.9 mmol/L and 2-hour postprandial glucose <7.8 mmol/L) or IGT (fasting plasma glucose <7 mmol/L and 2-hour postprandial glucose 7.8–11.0 mmol/L) or DM (fasting plasma glucose ≥7.0 mmol/L and/or 2-hour postprandial glucose ≥11.1 mmol/L) according to World Health Organization (WHO) diagnostic criteria. The study was approved by the Ethics Committee of our institution. The protocol was conducted in accordance with the Declaration of Helsinki. Written informed consent was obtained from all patients before being enrolled in the study.

### 2.2. Diagnosis of PPGL

According to the procedures described in the pertinent guidelines [10], the diagnosis of PPGL was based on clinical characteristics, plasma-free MNs, visualization of the tumor by computed tomography, magnetic resonance imaging, and/or 18F-FDG-PET/CT imaging. The diagnosis was confirmed histopathologically.

### 2.3. Hormonal and Biochemical Measurements

Fasting (at least 8 h) blood samples were collected in the early morning (7 am. to 9 am). The hormones required for the diagnosis of PPGL were assayed. Plasma-free MNs, including MN and NMN, were quantified by liquid chromatography-mass spectrometry (LC-MS/MS). Blood samples for measurement of fasting plasma total GLP-1 were collected before the start of alpha/beta blockade treatment, and a dipeptidyl peptidase-4 (DPP-4) inhibitor was added immediately after sampling. Then, plasma total GLP-1 concentrations were measured by a commercial ELISA kit (Millipore total ELISA). Basic laboratory tests, such as plasma glucose, lipid profile, and serum creatinine, were measured by standard methods in our institutional laboratory.

### 2.4. Statistical Analysis

Normally distributed data were presented as the mean ± standard deviation and were compared by independent *t* test. Non-normally distributed data are presented as the median and interquartile range (IQR) and were compared by the Kruskal–Wallis test. Relationships were examined using Pearson's correlation analysis or Spearman's signed rank correlation test. Multivariate analysis was performed using linear regression, adjusting for potential confounders. Differences in preoperative and postoperative GLP-1 and MNs levels were analyzed using paired Wilcoxon and paired *t*-tests. A *p* value <0.05 was considered statistically significant. All analyses were performed using SPSS 22.0 (IBM Corp., Armonk, NY, USA).

## 3. Results

### 3.1. Patient Characteristics

A total of 30 patients (13 men and 17 women) with PPGL were analyzed (Table 1). They were diagnosed with PCC (*n* = 23) or paraganglioma (*n* = 7) and received surgical treatment of the tumor. Of the thirty patients, the mean age was 40.0 ± 14.5 years. Among the 23 patients with PCC, 21 (91.3%) had unilateral localization, and two patients (8.7%) had bilateral localization. The overall median size of the largest tumor was 4.0 (3.4–5.5) cm. Twenty-five patients (83.3%) presented with adrenergic symptoms, including persistent hypertension (*n* = 16), palpitations (*n* = 14), and headaches (*n* = 15). Five patients (16.7%) were found incidentally on imaging. Twenty-nine patients in our cohort received alpha blockade prior to surgery, with the majority (60%) also receiving beta-adrenergic blockers. The most common comorbidities included hyperglycemia and dyslipidemia. Nine patients (30%) had abnormal lipids. Sixteen (53.3%) patients had hyperglycemia, and ten (33.3%) were diagnosed with DM. Seven patients were medicated with insulin during hospitalization, and none of them used GLP-1 agonists or DPP-4 inhibitors. The median fasting blood glucose level was 6.1 mmol/L (IQR, 4.9–6.6 mmol/L).

### 3.2. Association of Total GLP-1 Levels with MNs and Metabolic Risk Factors

**Figure 1:**
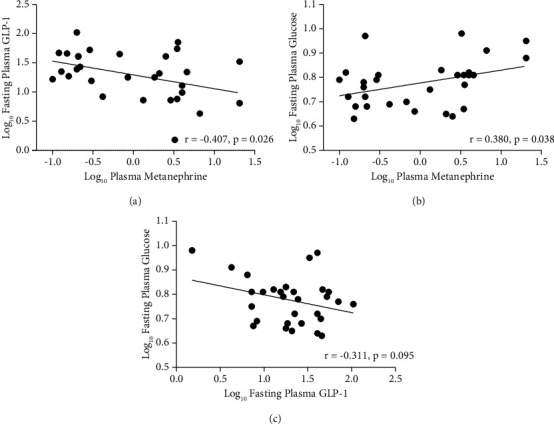
Pearson's correlation analysis among fasting plasma GLP-1, plasma MN, and plasma glucose levels. (a) Association between plasma metanephrine and fasting plasma GLP-1. (b) Association between plasma metanephrine and fasting plasma glucose. (c) Association between fasting plasma GLP-1 and fasting plasma glucose. Data were logarithmically transformed to approximate a normal distribution.

As a preliminary analysis, Pearson's or Spearman's correlation analysis was performed to evaluate associations between fasting plasma total GLP-1 levels, metabolic risk factors, and elevated plasma MN and NMN. A significant negative correlation was observed between basal levels of GLP-1 and elevated plasma MN (*r* = −0.407, *p*=0.026) (Figure 1(a)) but not with NMN (*r* = 0.029, *p*=0.877). We also found a positive correlation between fasting glucose levels and plasma MN (*r* = 0.380, *p*=0.038) (Figure 1(b)) and NMN levels (*r* = 0.450, *p*=0.013). While in PPGL patients, we did not find significant correlation of basal circulating GLP-1 levels with metabolic risk factors, such as age (*r* = −0.183, *p*=0.332), BMI (*r* = 0.273, *p*=0.144), waist circumference (*r* = 0.076, *p*=0.688), blood pressure (SBP: *r* = 0.071, *p*=0.709; DBP: *r* = 0.033, *p*=0.861), lipid profiles (TG: *r* = 0.171, *p*=0.365; LDL-C: *r* = −0.087, *p*=0.648; HDL-C: *r* = −0.158, *p*=0.406), or fasting glucose (*r* = −0.311, *p*=0.095), which indirectly suggested that the negative correlation between plasma MN and basal circulating GLP-1 concentration was independent of metabolic risk factors. However, this cannot be ruled out as the reason for the small sample size, as although the correlation was not significant, it can be seen from Figure 1(c) that there was a negative correlation between fasting blood glucose and GLP-1 levels. Therefore, we further evaluated the correlation between fasting plasma GLP-1 and MN by multiple linear regression, adjusting for potential confounders.

Linear regression analysis revealed that fasting total GLP-1 concentration was negatively associated with plasma MN (Table 2). In the multivariate model, adjusted for age, sex, BMI, serum creatinine, and the presence of hyperglycemia, the inverse associations between GLP-1 level and plasma MN remained significant (*β* = −0.493, *p*=0.013).

### 3.3. Effect of PPGL Resection on MN, NMN, and GLP-1 Levels

A subgroup of 13 patients with PPGL was examined after tumor removal. The median follow-up period was 4.9 months. Postoperative GLP-1 levels and plasma MN and NMN levels were evaluated for the effect of PPGL resection.  Table 3 shows plasma GLP-1 levels and the levels of MN/NMN before and after surgery. The Wilcoxon signed-rank test indicated that plasma MN and NMN levels decreased significantly after the operation, reflecting a successful surgery. For these patients, the mean fasting levels of total GLP-1 increased significantly after surgery from 25.81 to 40.3 pmol/L (*p*=0.017).

## 4. Discussion

This is the first study to link MNs with fasting plasma GLP-1 levels in PPGL patients. We report herein that the basal circulating level of GLP-1 was negatively correlated with plasma MN and that the fasting plasma GLP-1 level significantly increased after tumor removal.

Metabolic disorders in patients with PPGL have been described in previous reports [24–28]. Both impaired insulin secretion and increased insulin resistance have been implicated as underlying causes of glucose homeostasis in PPGL patients [29–31]. In patients with PPGL, the elevation of catecholamines has been shown to inhibit insulin secretion from islet *β* cells [32–34]. The activation of *α*2-AR in pancreatic *β*-cells is known to suppress insulin secretion in vivo and has been considered a mediator of the inhibitory effects of catecholamines in early-phase insulin release [35]. In addition, it has been reported that the potential explanation for insulin resistance observed in PPGL patients may be based on the presence of increased hepatic glucose production, decreased glucose uptake in the peripheral tissues, elevated free fatty acids, and lower adiponectin levels [25, 36, 37]. In the present study, we found a significant positive correlation between fasting blood glucose and high MNs, including MN and NMN, metabolites of epinephrine, and norepinephrine, respectively. Recently, Petrák and coworkers found impaired GLP-1 secretion during a dynamic meal test in patients with PHEO [17]. They found reduced postprandial levels of bioactive GLP-1 in patients with PHEO, which may contribute to impaired glucose metabolism. However, in their study, basal levels of GLP-1 were not changed by tumor removal, and they did not find a correlation between GLP-1 and excess catecholamines or MNs. Our study focused more on the effects of long-term catecholamine overproduction on basal circulating GLP-1 concentration. Reduced postprandial GLP-1 concentrations were observed in diabetic patients, but the importance of fasting plasma GLP-1 in altered metabolic outcomes has been questioned; both no differences and increased concentrations of fasting plasma total GLP-1 have been observed in diabetic patients compared with NGT, according to previous studies [21–23]. Therefore, evaluating the correlation of catecholamines with fasting GLP-1 levels may reduce the interference of metabolic risk factors compared with postprandial GLP-1. Additionally, as active GLP-1 is rapidly degraded by DPP-4 enzymes almost immediately on its release, we detected plasma total GLP-1 concentrations, which include any intact, active hormone as well as the inactive primary metabolite of GLP-1, in PPGL patients [38, 39]. In the present study, we found that basal circulating GLP-1 levels in PPGL patients increased after tumor removal. In addition, the mean level of circulating GLP-1 in PPGL patients was 25.81 pmol/L, and the published values for GLP-1 concentrations in a clinical study showed that the mean fasting levels of total GLP-1 in healthy subjects were approximately 44 pmol/L with the Millipore total ELISA [40, 41]. Combined with GLP-1 changes before and after surgery, it is reasonable to believe that fasting plasma total GLP-1 concentrations in PPGL patients decreased, despite the absence of healthy controls. These findings suggest that long-term overproduction of catecholamines appears to induce suppression of GLP-1 production in patients with PPGL. As we know, circulating free MNs could reflect tumor tissue catecholamine content and it can be used to distinguish catecholamine biochemical phenotype. High plasma-free MN indicates adrenergic (epinephrine-producing) biochemical phenotype tumors and noradrenergic phenotype usually lack epinephrine production [6]. In our study, we found an inverse association between the basal circulating GLP-1 level and plasma MN, which remained significant after adjustment for potential confounders such as age, sex, BMI, dysglycemia, and serum creatinine, suggesting that the decreased GLP-1 levels observed in PPGL patients may be related to an epinephrine-producing biochemical phenotype with chronic overproduction of epinephrine in vivo. Studies with larger sample sizes are needed to investigate GLP-1 levels in different catecholamine phenotypes.

A few previous experimental studies on the interaction between catecholamines and GLP-1 suggest that there are differences in the effects of epinephrine and norepinephrine on GLP-1 secretion due to different affinities for respective adrenergic receptors. These studies have shown that epinephrine increases GLP-1 secretion from isolated vascularly perfused rat ileum and GLUTag cells due to the combined activation of the three types of receptors. Activation of *β*- and *α*1-AR positively regulates GLP-1, while overexpression of *α*2A-AR suppresses adrenaline-induced GLP-1 exocytosis [14, 15]. Unlike epinephrine, norepinephrine inhibits GLP-1 secretion via its action on adrenergic *α*-ARs [16]. Interestingly, we found that the basal level of GLP-1 in PPGL patients was negatively correlated with plasma MN, the metabolic product of epinephrine, but not with NMN. This is similar to glucagon secretion in response to epinephrine. In isolated rat islets, epinephrine exerts both inhibitory and stimulatory effects on glucagon secretion, depending on receptor occupancy. The findings of known adrenergic receptor downregulation and desensitization in PPGL patients with long-term catecholamine excess might lead to clarification of the mechanism. In an in vitro study, observations supported the hypothesis that neurotransmitters stimulate the secretion of intestinal enteroendocrine cells only over brief time periods [15]. To some extent, this explains our finding that GLP-1 secretion may be inhibited in humans with long-standing exposure to high catecholamine levels, and chronic epinephrine overproduction is likely to inhibit rather than stimulate GLP-1 release. Furthermore, glucose metabolism disorders might have added another layer of complexity to the decrease in GLP-1 levels.

Several limitations should be acknowledged in this study. First, the sample size was small; larger sample studies are required to further validate our findings. Besides, further experimental studies are needed to determine the mechanism behind our results. In addition, we found a correlation between GLP-1 and MN; however, it remains to be determined whether GLP-1 levels are directly caused by catecholamine in PPGL patients.

## 5. Conclusion

In conclusion, we found decreased fasting total GLP-1 concentrations in patients with PPGL and that GLP-1 levels were negatively associated with plasma MN. Our findings suggest that long-term overproduction of catecholamines appears to induce suppression of GLP-1 production. Further studies are required to elucidate the mechanism of GLP-1 secretion with chronic catecholamine exposure and to further understand the role of basal circulating GLP-1 in metabolic regulation under stress in vivo.

## Figures and Tables

**Table 1:** Clinical characteristics of patients with PPGL (*n* = 30).

Characteristics	
Age (years)	40.4 ± 14.5
Female, *n* (%)	17 (56.7%)
Body mass index (kg/m^2^)	21.54 ± 3.42
Waist circumference (cm)	80.88 ± 10.52
PCC unilateral, *n* (%)	21(91.3%)
PCC bilateral, *n* (%)	2 (8.7%)
Extra-adrenal (PGL), *n* (%)	7 (23.3%)
Tumor diameter (cm)	4.0 (3.4–5.5)
Asymptomatic, *n* (%)	5 (16.7%)
Symptomatic, *n* (%)	25 (83.3%)
Headache, *n* (%)	15 (50%)
Palpitations, *n* (%)	14 (46.7%)
Persistent hypertension, *n* (%)	16 (53.3%)
Beta-blockers, *n* (%)	18 (60%)
Alpha-blockers, *n* (%)	29 (96.7%)
Hyperglycemia, *n* (%)	16 (53.3%)
Dyslipidemia, *n* (%)	9 (30%)
Fasting plasma glucose (mmol/L)	6.1 (4.9–6.6)
Fasting plasma total GLP-1 (pmol/L)	27.8 ± 14.7
DPP-IV inhibitors or GLP-1 agonists, *n* (%)	0
Insulin therapy, *n* (%)	7 (23.3%)
Triglyceride (mmol/L)	1.31 ± 0.67
LDL-C (mmol/L)	3.17 ± 0.79
HDL-C (mmol/L)	1.37 ± 0.32
Serum creatinine (umol/L)	66.5 (56.0–74.3)
Plasma metanephrine (nmol/L)	1.09 (0.21–3.66)
Plasma normetanephrine (nmol/L)	9.11 (4.23–20.56)

Data are expressed as mean ± SD or median (internal quartile range (IQR)). PPGL: pheochromocytoma and paraganglioma; PCC: pheochromocytoma; PGL, paraganglioma; LDL-C, low-density lipoprotein cholesterol; HDL-C, high-density lipoprotein cholesterol; GLP-1, glucagon-likepeptide-1.

**Table 2:** Multiple linear regression analyses for the association of plasma GLP-1 levels with plasma-free MN.

	Regression analysis			
	Unadjusted coefficient *β*(95% CI)	*p*	Adjusted coefficient *β*(95% CI)	*p*
Plasma MN	−0.407 (−0.444–−0.031)	0.026^*∗*^	−0.493 (−0.508–−0.067)	0.013^*∗*^

Multivariate analysis was performed by linear regression adjusting for potential confounders such as age, gender, BMI, hyperglycemia, and serum creatinine. Data were logarithmically transformed to approximate a normal distribution. MN: metanephrine.

**Table 3:** Plasma MNs and GLP-1 levels of study patients before and after surgery (*n* = 13).

	Before surgery	After surgery	*p*
Plasma MN (nmol/l)	0.30 (0.15–2.84)	0.12 (0.08–0.21)	0.007^*∗*^
Plasma NMN (nmol/l)	8.42 (4.51–20.56)	0.48 (0.43–0.56)	0.001^*∗*^
Fasting total GLP-1 (pmol/l)	25.81 ± 15.83	39.01 ± 19.56	0.017^*∗*^

The significance of differences between preoperative and postoperative values was analyzed using paired Wilcoxon or paired *t*-tests. Data are shown as mean ± SD or the median (internal quartile range (IQR)). GLP-1: glucagon-likepeptide-1; MN: metanephrine; NMN: normetanephrine.

## Data Availability

All the data generated or analyzed during this study are included in this article. Further enquiries can be directed to the corresponding author.

## Abbreviations

PPGL: Pheochromocytoma/paraganglioma
PCC: Pheochromocytoma
PGL: Paraganglioma
GLP-1: Glucagon-like peptide-1
T2D: Type 2 diabetes
MNs: Metanephrines
MN: Metanephrine
NMN: Normetanephrine
BMI: Body mass index
PHEO: Pheochromocytoma
AR: Adrenergic receptor
DM: Diabetes mellitus
BMI: Body mass index
WC: Waist circumference
SBP: Systolic blood pressure
DBP: Diastolic blood pressure
LDL-C: Low-density lipoprotein cholesterol
HDL-C: High-density lipoprotein cholesterol.
